# Constrained IoT-Based Machine Learning for Accurate Glycemia Forecasting in Type 1 Diabetes Patients

**DOI:** 10.3390/s23073665

**Published:** 2023-03-31

**Authors:** Ignacio Rodríguez-Rodríguez, María Campo-Valera, José-Víctor Rodríguez, Alberto Frisa-Rubio

**Affiliations:** 1 Departamento de Ingeniería de las Comunicaciones, Universidad de Málaga, 29010 Málaga, Spain; 2 Departamento de Tecnologías de la Información y las Comunicaciones, Universidad Politécnica de Cartagena, 30202 Cartagena, Spain; 3 CIRCE—Centro Tecnológico (Research Centre for Energy Resources and Consumption), Av. Ranillas, Edf. Dinamiza 3D, 50018 Zaragoza, Spain

**Keywords:** constrained devices, diabetes, IoT, monitoring, machine learning

## Abstract

Individuals with diabetes mellitus type 1 (DM1) tend to check their blood sugar levels multiple times daily and utilize this information to predict their future glycemic levels. Based on these predictions, patients decide on the best approach to regulate their glucose levels with considerations such as insulin dosage and other related factors. Nevertheless, modern developments in Internet of Things (IoT) technology and innovative biomedical sensors have enabled the constant gathering of glucose level data using continuous glucose monitoring (CGM) in addition to other biomedical signals. With the use of machine learning (ML) algorithms, glycemic level patterns can be modeled, enabling accurate forecasting of this variable. Constrained devices have limited computational power, making it challenging to run complex machine learning algorithms directly on these devices. However, by leveraging edge computing, using lightweight machine learning algorithms, and performing preprocessing and feature extraction, it is possible to run machine learning algorithms on constrained devices despite these limitations. In this paper we test the burdens of some constrained IoT devices, probing that it is feasible to locally predict glycemia using a smartphone, up to 45 min in advance and with acceptable accuracy using random forest.

## 1. Introduction: The Forecasting Problem in Type 1 Diabetes Mellitus

Due to insulin deficiency, diabetes causes elevated blood sugar. Glycemic homeostasis is a closed-loop process that regulates blood sugar in healthy people. Thus, the pancreas produces insulin from β cells that are sensitive to high glucose levels and enables glucose to enter cells, reducing hyperglycemia.

People with type 1 diabetes mellitus (DM1) cannot naturally regulate their insulin. DM1 patients who do not make insulin must inject or hold an insulin pump to lower their glycemic levels. Diabetics must also monitor their glucose levels multiple times a day and use these data and other variables, such as meals, exercise, and others, to anticipate their glycemia. Then, they must determine how much insulin they need to maintain normal blood glucose levels (avoiding both hyper- and hypoglycemia). Thus, precise blood glucose prediction is crucial for insulin dosing [1].

Thankfully, modern technology opens up new diabetes control alternatives. Continuous glucose monitoring is now essential to diabetes management (CGM) [2]. This notion has revolutionized diabetes management by continually providing glucose level magnitude, tendency, frequency, and duration. CGM-based devices can take one glucose measurement every minute (1440 day) and sometimes also measure other variables concurrently [3]. Such data might improve glucose level prediction [4,5].

There are alternative methods to continuous glucose monitoring (CGM) devices that can provide 24 h patient monitoring [6], which allows for the collection of essential health data to aid in developing effective glycemia treatments. Specific variables, such as heart rate, temperature, sleep quality, and exercise, can be reliably monitored using the primary sensors found in commercially available smartwatches [7].

Telemedicine, used to monitor patients remotely, employs a wireless body area network (BAN) comprising wearable computing units [8]. BANs need to incorporate all functions that are either inside, outside, or close to the human form, along with communication capabilities. BANs are well-suited for observing patients’ biological signals in a medical setting. A wireless body area network (WBAN) is created as a wireless network formed with sensors that utilize a certain number of networks and wireless appliances that remotely record biosignals in diverse circumstances. BANs mandate a body gateway and a network hub, usually in the form of a smartphone.

However, current diabetes management body gateways [9] do not analyze biometric data locally or predict blood sugar concentrations [10]. The data are transferred to an adjacent gateway device, which then sends it to the cloud for enhanced analysis [11,12]. Since glycemia can change rapidly in DM1 patients and unforeseen changes in their daily routine can occur, continuous monitoring of data is essential to predict future blood glucose levels, and local computing and forecasting of glycemia may be necessary in the event of lack of connection.

The study discussed here assumes that wearable devices can execute forecasting algorithms that can interpret CGM sensor data in real time and create DM1 patient alarms without cloud infrastructure, thus conserving battery power and memory. Researchers and practitioners are increasingly interested in merging IoT constrained devices and using them to compute.

In this paper, we investigate the feasibility of predicting glycemic levels in individuals with diabetes mellitus type 1 (DM1) using wearable IoT devices and machine learning algorithms. Specifically, we aimed to determine whether local processing and analysis of continuous glucose monitoring (CGM) sensor data using lightweight machine learning algorithms could achieve accurate and timely glycemia predictions, without relying on cloud infrastructure.

We collected CGM sensor data from DM1 patients using wearable IoT devices, which provided continuous glucose measurements every minute. We then used machine learning algorithms, specifically random forest, to model glycemic level patterns and make predictions up to 45 min in advance. To overcome the computational limitations of wearable IoT devices, we implemented lightweight machine learning algorithms and performed preprocessing and feature extraction to reduce the computational burden.

In the context of diabetes management, predicting future glycemic levels is a critical task that enables patients to adjust their insulin dose and take necessary actions to prevent hypo- or hyperglycemia. Predictive models leverage past glucose readings, along with other relevant data such as meals, physical activity, and insulin doses to forecast future glucose levels. These models can be based on statistical methods, machine learning algorithms, or a combination of both.

The prediction process generally involves several steps, including data preprocessing, feature engineering, model selection, and model training. Data preprocessing involves cleaning and transforming the raw data to ensure its quality and consistency. Feature engineering involves selecting and transforming relevant features from the preprocessed data to feed into the prediction model. Model selection involves choosing an appropriate algorithm or ensemble of algorithms that best fits the data and the prediction task. Model training involves fitting the selected model to the training data, evaluating its performance, and fine-tuning its parameters to optimize its predictive accuracy.

When selecting a prediction algorithm, several characteristics must be taken into account, such as model complexity, interpretability, scalability, and generalization ability. Model complexity refers to the number of parameters and computations required to fit the model to the data. A more complex model may result in better prediction accuracy, but it may also be prone to overfitting and may require more computational resources to train and deploy. Interpretability refers to the ability to understand and explain how the model makes predictions, which is critical for clinical decision making and trust in the model’s outputs. Scalability refers to the ability to handle large datasets and generalize well to new data. Generalization ability refers to the ability of the model to make accurate predictions on unseen data, which is crucial for clinical applicability and reliability.

In the context of wearable devices and IoT, additional factors such as energy efficiency, memory footprint, and real-time processing capabilities must also be considered when selecting a prediction algorithm. Machine learning algorithms that are lightweight, require low computational resources, and operate in real-time can be ideal for predicting glycemic levels on wearable devices. For instance, random forest and support vector machine (SVM) are often used for glucose level prediction tasks in diabetes management due to their good performance and relatively low computational cost.

Local analysis and forecasting may have interesting advantages, including that it may be performed without an internet connection, which may be unavailable in distant areas, during phone coverage failures, or if the unit is in flight mode and unable to acquire data from the cloud (theatre, flight, or lessons). Additionally, local processing enhances data control. Since wearable device data are not sent to the cloud, we can give users full control over their data. This supports this research’s objective of data privacy. Smart healthcare systems need data privacy.

Previous studies have used several approaches to anticipate glycemia, some of which are better suited to a particular computing environment [13,14]. As more factors are included, data processing needs will increase.

In this paper, we first introduced the problem of forecasting blood sugar levels in individuals with type 1 diabetes mellitus (DM1) and its importance in managing the condition. Section 2 provides a review of previous works and the related literature, offering context and background information on the subject. Section 3 presents various methods for glucose level prediction, including the techniques and algorithms used. In Section 4, we detail the monitoring campaign undertaken to collect data for the study. Section 5 describes the implementation of the selected techniques, including the hardware, software, and configurations. Section 6 presents the results and discusses their implications, comparing the performance of different approaches. Finally, Section 7 concludes the paper, summarizes the findings, and suggests future directions for research in this area.

## 2. Previous Works and Literature Review

Constrained devices, such as smartphones and low-power electronics, have become increasingly popular in biomedical applications due to their cost-effectiveness, portability, and ease of use. These devices have been used for a wide range of biomedical applications, such as disease detection, diagnosis, and monitoring. In recent years, researchers have explored new techniques to enhance the capabilities of these devices while minimizing their power consumption.

One of the key challenges in using constrained devices in biomedical applications is to avoid developing demanding algorithms that can operate on limited resources. Shoaran et al. [15] proposed an energy-efficient classification method for resource-constrained biomedical applications. The proposed method utilizes a combination of optimized feature extraction and machine learning algorithms to reduce power consumption while maintaining high classification accuracy. The results demonstrated that the proposed method achieved an F1 score of 99.23% with a 27× reduction in energy-area-latency product.

In addition to energy-efficient algorithms, wireless power transfer systems have also been developed to enable long-term operation of biomedical devices without the need for battery replacement. Ahire and Gond [16] presented a review of wireless power transfer systems for biomedical applications. The authors discussed various wireless power transfer techniques, including electromagnetic induction and radio frequency energy harvesting and their potential applications in biomedical devices.

Smartphones have also emerged as a popular platform for developing low-cost and portable biomedical devices. Banik et al. [17] provided a review of recent trends in smartphone-based detection for biomedical applications. The authors discussed various smartphone-based sensors, such as cameras, microphones, and accelerometers, and their potential applications in biomedical fields including disease detection and diagnosis.

Overall, the use of constrained devices in biomedical applications has shown great potential in improving healthcare accessibility and reducing costs. With continued advancements in energy-efficient algorithms, wireless power transfer systems, and smartphone-based sensors, the future of constrained devices in biomedical applications looks promising [18].

Computational effort is a significant challenge in executing machine learning algorithms. Machine learning algorithms require a large amount of data and complex mathematical computations to train models and make predictions. The size of the datasets used in machine learning applications has significantly increased in recent years, and as a result, the computational resources required to process these data sets have also increased.

The computational effort required to execute machine learning algorithms can be affected by several factors, including:

- Model complexity: More complex models, such as deep neural networks, require more computational resources to train and execute.
- Size of the dataset: Larger datasets require more computational resources to process.
- Hardware resources: The hardware resources available, such as CPU, GPU, and memory, can affect the speed and efficiency of machine learning algorithms.

Overall, the computational effort problem in executing machine learning algorithms is a significant challenge, but researchers and practitioners are continuously developing new techniques to address this challenge and make machine learning more efficient and scalable.

Performing the task of forecasting glycemia requires a specific type of hardware. Given that this task will be performed multiple times per hour, “on-the-fly” style, with the model being recalculated and predicted each time, it is crucial to consider the execution time. The computational effort required is a significant constraint, and to the authors’ knowledge, no previous work in diabetes management has studied or compared the computational time and accuracy of prediction methods to achieve a compromise solution.

Depending on the machine learning (ML) algorithm selected, we may face computer limitations, not just due to the hardware, but also due to the software, including the operating system [19]. This idea of device restrictions has been previously explored in more demanding processes [20]. In that case, classification methods were studied and compared.

A comparison of ML performance in medicine can be found in ref. [21] with functional magnetic resonance imaging (fMRI), where the accuracy of six different ML algorithms (including RF and SVM) applied to the neuroimaging data of individuals responding to a range of propositional statements was compared. RF was found to be more accurate than SVM in this interesting study. Each algorithm’s performance was assessed by reducing the feature set, reducing the amount of data to handle, and thus reducing the computational effort without sacrificing accuracy.

In ref. [22], we find a general comparison of ML algorithms, including RF and SVM. In this case, the algorithms were tested using the Gisette dataset, a free dataset for handwritten digit recognition problems. SVM was one or multiple orders of magnitude faster, while RF was more precise.

Some other approaches can be interesting, including novel approaches such as the augmented Lagrangian method (ALM), one of the algorithms in a class of methods used for constrained optimization of nonlinear problems (NLP) [23], which is applied in fields such as optimal economic growing quantities for reproductive farmed animals [24] and customer credit [25] and could also be applied to our problem.

In 2020, a study [26] presented different models of glycemia dynamics for improved management of type 1 diabetes mellitus using advanced intelligent analysis in an Internet of Things (IoT) context. The authors proposed a new model for glycemia dynamics that takes into account the dynamics of both insulin and glucose levels. They compared this model with other existing models, including the well-known Bergman’s minimal model, and evaluated the performance of the models using data collected from a clinical trial. The results showed that the proposed model outperformed the other models in terms of accuracy and efficiency. We affirm that random forest (RF), as both a predictive algorithm and FS strategy, offered the best average performance (root median square error, RMSE = 18.54 mg/dL) throughout the 12 considered predictive horizons (up to 60 min in steps of 5 min), showing that support vector machines (SVM) had the best accuracy as a forecasting algorithm when considering, in turn, the average of the six FS techniques applied (RMSE = 20.58 mg/dL).

In 2021, another study [27] compared different feature selection and forecasting machine learning algorithms for predicting glycemia in type 1 diabetes mellitus. The authors used a dataset of glucose readings collected from patients with type 1 diabetes and compared the performance of different feature selection techniques, including PCA and Lasso, with different machine learning algorithms, including random forest, SVM, and LSTM. The results showed that random forest and SVM with PCA feature selection outperformed the other techniques in terms of prediction accuracy, whereas LSTM did not perform as well. The authors also evaluated the interpretability of the models and concluded that the Bayesian regularized neural network (BRNN) offered the best performance (0.83 R^2^) with a reduced root median squared error (RMSE) of 14.03 mg/dL.

The authors have not found any data on computational effort in glucose prediction for DM1. Therefore, this work’s primary goal was to improve the computational performance of various glycemia forecasting methods. Then, we will analyze the possibility of running these algorithms on small devices. Our findings could be applied to other similar ML applications in time series data frequently used in medicine.

Using machine learning algorithms on constrained devices, such as wearable devices, smartphones, and Internet of Things (IoT) devices, is challenging. These devices generate a large amount of data, but their limited computational resources, including processing power, memory, and battery life, make it difficult to efficiently execute these algorithms. Model optimization techniques, such as model compression and pruning, can be used to reduce the size and complexity of machine learning models. Specialized hardware, such as GPUs and ASICs, can also be used to accelerate the execution of machine learning algorithms. However, despite these optimizations, the problem of computational effort in constrained devices remains a significant challenge, and further research is required to develop new techniques and algorithms that are specifically designed to run efficiently on these devices. Edge computing is a promising approach that can reduce the need for data transmission and improve the efficiency of machine learning algorithms on constrained devices.

Constrained devices are electronic devices with limited resources, such as computing power, memory, and battery life. They are designed for specific tasks and typically have small form factors and low power consumption, making them suitable for use in various Internet of Things (IoT) applications and embedded systems [28]. The Internet of Medical Things (IoMT) is an ecosystem of connected medical devices and sensors that help healthcare providers to gather, analyze, and act on patient data in real time. Constrained devices play a crucial role in this ecosystem by providing the means to collect, process, and transmit medical data from remote and often challenging environments.

Examples of constrained devices include smartphones, wearable devices, smart sensors, and embedded systems in automobiles, appliances, and medical devices. These devices often operate in environments with limited network connectivity, low processing power, and limited storage.

To accommodate the limited resources of constrained devices, engineers must consider the device’s constraints when designing software and applications. This requires a different approach to software development compared to traditional desktop or mobile applications. Constrained devices typically use lightweight protocols and data formats, such as message queuing telemetry transport (MQTT) [29] and constrained application protocol (CoAP) [30], and usually rely on edge computing or cloud computing to perform some processing tasks.

MQTT is a publish/subscribe messaging protocol that is optimized for low-bandwidth and high-latency networks. It allows devices to efficiently exchange messages with a server, known as a broker, while minimizing the use of resources.

CoAP is a RESTful web transfer protocol that is designed for use with constrained devices and networks. It is a highly efficient protocol with a small code footprint and low network overhead.

Both MQTT and CoAP are designed to minimize the use of resources and provide reliable communication between devices, even in challenging network conditions. This makes them well-suited for use in constrained devices, which often have limited computing power [31], memory, and network connectivity.

With these limitations, we focused on the challenge of executing ML algorithms locally, considering the limited computational power. However, there are several strategies for running machine learning algorithms on constrained devices, despite these limitations.

One approach is to perform some or all of the processing on a more powerful device, such as a server or cloud-based system. Another strategy is to perform preprocessing on the data collected by the constrained device [32], reducing the amount of data that needs to be transmitted [33] to the more powerful device for analysis.

Another approach is to use lightweight machine learning algorithms that have a smaller computational footprint. These algorithms should be designed to be fast, efficient, and require fewer resources to run than more complex algorithms, such as deep neural networks. This makes them well-suited for use on constrained devices.

## 3. Methods for Glucose Level Prediction

**Figure 1:**
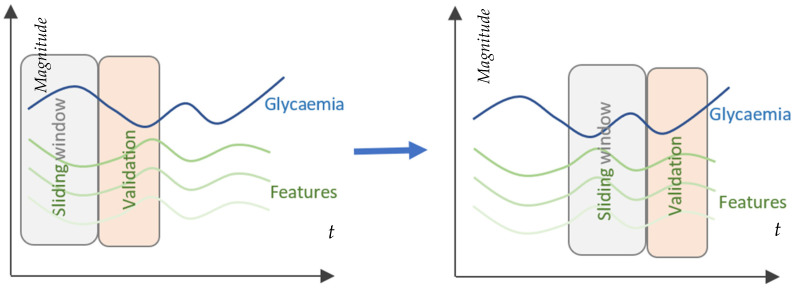
Cross-validation and time series analysis using a slide window.

Figure 1 depicts the fragmentation of acquired data into input windows for the patient-centered prediction model. Every 5 min, a single measurement is taken to sample the CGM sensor’s data. The sampled values are used to create a moving window for the past (PSW), which contains historical values of the last 6 h. The PSW regulates the quantity of data used by the model for prediction. On the basis of the data from the moving window, the model continually forecasts glucose levels 15 and 45 min prior to the present time at predetermined prediction horizons (PH).

The sliding window operates as described. Every time a new CGM value is received, the training dataset is restructured by deleting the oldest observation, shifting all values up by one place and then inserting the newly arrived value as the newest value. Consequently, the size of the dataset and order of the observations are always maintained.

### 3.1. Feature Selection

Feature selection is an essential step in machine learning, which aims to identify the most relevant features or variables that can improve the accuracy of a predictive model. Feature selection techniques can be broadly categorized into filter, wrapper, and embedded methods. However, some of these methods can be computationally demanding, which can limit their applicability to resource-constrained devices.

Filter methods are computationally efficient and commonly used in feature selection. These methods evaluate the relevance of each feature independently of the model using statistical tests or correlation analysis. For example, the Chi-squared test [34] is a widely used statistical test in feature selection. These methods can efficiently identify the most relevant features but may not consider interdependence between features.

Embedded methods are another class of feature selection techniques that are computationally efficient and integrated with the learning algorithm. These methods aim to learn the best subset of features during the training process. For example, Lasso [35] and ridge regression are widely used embedded methods that can automatically select relevant features while simultaneously regularizing the model to prevent overfitting.

Wrapper methods are computationally demanding as they involve evaluating different subsets of features by repeatedly training and validating the model [36]. These methods can accurately select the most relevant features but may not be suitable for resource-constrained devices. For example, recursive feature elimination (RFE) is a popular wrapper method that recursively removes features and evaluates the model’s performance until the optimal subset of features is identified [37].

In summary, filter and embedded methods are computationally efficient and suitable for feature selection in resource-constrained devices. These methods can efficiently identify the most relevant features and improve the predictive accuracy of the model. On the other hand, wrapper methods may be computationally demanding but can provide accurate feature selection. Hence, the choice of feature selection technique depends on the available computational resources and specific requirements of the application.

We then used Lasso regression. It aims to minimize the cost function and automatically identifies the relevant features while discarding the redundant or irrelevant ones. This is achieved by setting the coefficient of the discarded feature to zero in the regression model.

### 3.2. Forecasting Algorithms

The glucose level prediction methods considered in this work are the following:

Random forest (RF): RF bagging is a method used by algorithms that is characterized by the repeated sampling of data instances in order to generate different training subsets based on the same training data [38]. Following the completion of each training subset, decision trees are generated, which are then compiled into an ensemble. The result of an incoming data instance class label is ultimately decided by a unit vote that is cast by each tree. The use of RF is versatile and needs just a small amount of processing resources.

In time series forecasting, random forest can be used to model the relationship between the past values of a time series and its future values [39]. To do this, the algorithm first divides the time series into training and testing sets. The training set is used to train the decision trees, while the testing set is used to evaluate the performance of the algorithm.

Each decision tree in the random forest model is trained on a random subset of the training data, and each tree is grown to a different depth. The final prediction is made by combining the predictions of all the trees using a majority voting mechanism. This process helps to reduce the variance and overfitting associated with individual decision trees.

Support vector regression (SVR) [40]: Support vector regression is a machine learning algorithm that is commonly used for regression tasks. It is computationally efficient, even for large datasets, and can be used for time series forecasting by converting the time series into a regression problem. It is a type of regression analysis that uses support vector machine (SVM) to model the relationship between the inputs and outputs.

In time series forecasting, SVR can be used to model the relationship between the past values of a time series and its future values. To do this, the algorithm first divides the time series into training and testing sets. The training set is used to train the SVM, while the testing set is used to evaluate the performance of the algorithm.

The goal of SVR is to find the best function that can accurately predict the output based on the inputs. This function is represented as a hyperplane in a high-dimensional feature space, and it is trained using a set of support vectors that define the hyperplane. The SVM algorithm optimizes the hyperplane such that it maximizes the margin between the hyperplane and the support vectors, while also ensuring that the prediction error is minimized.

Random forest and support vector regression (SVR) are generally considered to be computationally efficient algorithms that can be used for executing machine learning tasks on constrained devices like smartphones or Raspberry Pi.

Random forest is a decision tree-based algorithm that relies on the idea of constructing a multitude of decision trees at training time and outputting the class that is the mode of the classes (classification) or mean prediction (regression) of the individual trees. The algorithm can efficiently handle large datasets with high dimensionality, and it is known for being computationally efficient compared to other algorithms, such as neural networks, which require significant computational resources.

Similarly, SVR is also known for its ability to handle high-dimensional data and for being computationally efficient. It is based on the idea of mapping input vectors into a high-dimensional feature space using a kernel function, which allows the algorithm to efficiently find a linear regression function in that feature space. The algorithm can handle non-linear data and is known to provide good results with relatively small datasets.

On the other hand, long short-term memory (LSTM) is a type of recurrent neural network (RNN) that is often used for processing sequential data, such as time series data. LSTMs can be computationally intensive, requiring a significant amount of processing power and memory to run effectively. This makes them less suitable for constrained devices such as smartphones or Raspberry Pi, which have limited computational resources.

In summary, while random forest and SVR are computationally efficient and can be used for executing machine learning tasks on constrained devices, LSTMs may not be the best choice due to their high computational demands.

The measure of predictive performance, the root mean square error (RMSE), is a metric that is used in the analysis of prediction accuracy. This is the most commonly used metric to measure prediction performance in the related literature.

All of the predictive tasks were optimized using hyper-tunning according to Table 1.

To develop a proper machine learning model, several steps need to be taken, including data preparation, model training, and model evaluation. The following actions were taken in the given scenario:

- Transformation: The first step is to consider the parameters that will be used as input to the models. It is important to select the most representative features for the model, and data transformation can help achieve this. For example, feature scaling or log transformation can be used to normalize and standardize the data.
- Normalization: After transformation, the next step is to normalize the data. Normalization helps to bring all the values within a specific range. In this case, the normalization process was applied to scale all the values between 0 and 1.
- Evaluating metric: To evaluate the performance of the model, root mean square error (RMSE) and R squared (R^2^) metrics were selected. RMSE provides an estimate of the mean of the error found, while R squared describes the standard deviation of the fit. These metrics can help to determine how well the model is performing and if it needs to be adjusted or retrained.
- Validation method: 10-fold cross-validation and 5 repetitions over the training dataset were used as the validation method. Cross-validation is a technique used to evaluate the performance of the model by splitting the dataset into training and testing sets. The 10-fold cross-validation method involves dividing the dataset into ten equal parts, using nine of them for training and the remaining one for testing. This process was repeated five times to ensure the stability and reliability of the results.
- Test: Finally, the model is tested to evaluate its performance on the new data. This step is important to determine if the model is able to generalize well on unseen data and can be used for prediction.

In summary, developing a proper machine learning model involves several steps, including data preparation, model training, and model evaluation. The steps listed in the given scenario include data transformation, normalization, selecting appropriate evaluation metrics, choosing a suitable validation method, and testing the model on new data.

One of the main contributions of this work compared to previous works is the comprehensive data collection approach, which combined data from multiple sources such as CGM devices, Fitbit Charge 5 smart bands, and other bio-medical devices. This allowed for a more detailed understanding of the factors affecting glycemic levels in type 1 diabetes patients. The study also employed the Lasso algorithm for variable selection, enabling a more focused and accurate prediction by considering only the most relevant variables. This approach helped identify key variables and their ranking, providing valuable insight into the factors affecting glycemia.

Another significant contribution is the use of sliding cross-validation for model building and later predictions, which takes into account the time window of past data and the predictive horizon. This method ensures more robust models and predictions. Furthermore, the study compared the performance and computational efficiency of two machine learning algorithms, random forest (RF) and support vector regression (SVR), in predicting glycemic levels. The comparison included both accuracy and computational efficiency, which is essential for real-time applications on devices with limited resources such as smartphones and Raspberry Pi devices.

## 4. Monitoring Campaign

To validate our proposal, a monitoring campaign was previously performed. The diabetic person monitoring system consisted of a portable Abbott Freestyle Libre paired with a smartphone, which could transfer data to the cloud. Software installed on the patient’s constrained device regularly connected to the CGM device and other biomedical devices through a secure NFC-based short-range wireless connection or Bluetooth Low Energy to obtain the most current measurements [41].

The CGM sensor can measure blood glucose (milligram/deciliter, mg/dL) every min [42] by inserting a sensor in the patient. With an average absolute relative subtraction (MARD) of 11.4%, the value of the CGM sensor is an estimation of the real sugar level in the blood [43,44], as specified by the manufacturer. The maximum lifespan of the CGM sensor is fourteen days. A total of 13,440 h of data was collected.

The dataset was completed using the Fitbit Charge 5 smart band. This type of wearable has experienced an important advance recently [45]. Each subject wore an intelligent watch that continuously recorded the movement performed (number of steps), heart rate, and minutes of sleep. Although they are not specialized medical gadgets, the inputs are legitimate and their energy usage is lowering.

The aforesaid technology was tested on 40 DM1 diabetics in 2021 by local hospitals. The research followed the Helsinki declaration. Table 2 lists the patients’ clinical features.

During monitoring, which is performed by passive means, patients were advised to adhere to their normal routine and consume a calorically balanced diet.

We considered the following features collected in our experimental phase:

- Glycemia. A set of past measurements.
- Insulin injections: Past values of rapid doses of insulin.
- Meal ingestion: In the same manner as insulin, previous values.
- Exercise: The number of steps representing influential past data.
- Heart rate: Current value as well as previous ones.
- Sleep: Only values for “asleep” or “awake” were gathered for the data. It makes sense to think of sleep as a compilation of all the times in the past that one has slept.

## 5. Implementation

Running machine learning (ML) algorithms on constrained devices like smartphones or Raspberry Pi can be a challenging task due to the limited hardware resources available. However, with the advancement of technology, it is now possible to run ML algorithms on such devices. In this regard, there are several technical considerations that need to be taken into account.

It is essential to select the appropriate ML algorithms that are optimized for running on constrained devices. Some algorithms require high computational resources and may not be suitable for such devices. Therefore, it is important to choose algorithms that have been specifically designed to run on these devices.

The selection of the programming language and development environment is critical. In general, high-level languages such as Python and R are preferred for ML tasks, and there are various libraries and frameworks available that support these languages. For instance, TensorFlow Lite and PyTorch Mobile are popular frameworks for running ML models on constrained devices. On the other hand, there are several development environments available, such as UserLAnd and Raspbian, that allow running ML algorithms on devices with different operating systems.

It is important to optimize the code for efficient execution on constrained devices. This can be accomplished by minimizing the memory usage and reducing the computation time. The code can be optimized by using techniques such as pruning, quantization, and compression, which can reduce the size of the models and improve their execution time.

Additionally, it is essential to consider the hardware limitations of the device when running ML algorithms. For instance, the memory capacity, processing speed, and storage space of the device need to be taken into account. In this regard, it may be necessary to use techniques such as data batching, which can reduce the memory usage by processing the data in smaller batches.

Running ML algorithms on constrained devices such as smartphones or Raspberry Pi requires careful consideration of the algorithms, programming language, development environment, code optimization techniques, and hardware limitations. By taking these factors into account, it is possible to develop efficient and effective ML models that can run on these devices.

We used R v4.2.2 software together with the CARET (Classification And REgression Training) tool version 6.0–93 to develop the initial models. In order to compare the performance of two constrained devices, namely a smartphone and a Raspberry Pi, we conducted several experiments.

The smartphone we employed for our experiments was a Samsung S22 model, equipped with an octa-core Snapdragon 8 Gen 1 system-on-chip consisting of 1 × 3.00 GHz Cortex-X2, 3 × 2.50 GHz Cortex-A710, and 4 × 1.80 GHz Cortex-A510, as well as 8 GB of LPDDR5 RAM running at 5500 MHz. To leverage the device’s internal storage of 128 GB UFS 3.1 for computation, we used UserLAnd, an open-source app that allows running several Linux distributions, as a Debian simulator. Despite the fact that the Samsung S22 runs on Android 13, we were able to run R and its libraries on UserLAnd. To ensure that the computations did not interfere with the device’s daily use, we reserved 7/8 cores for computation.

For the other device, we used a Raspberry Pi 4b. Raspberry Pi is an inexpensive device created to facilitate computing in developing countries. It is affordable and has sufficient capabilities that make it ideal for IoT development [46]. Raspberry Pi 4b leverages a Broadcom BCM2711 SoC with a 1.5 GHz 64-bit quad-core ARM Cortex-A72 processor and 8 GB LPDDR4 RAM running at 3200 MHz. We performed computations using a Micro SD HC 64GB UHS-3 Class 10. We ran the mathematical software R using Raspbian, a freeware system with based on Debian, which is specially conceived for Raspberry Pi device. In this case, we used all four cores to achieve the forecasting tasks.

## 6. Results and Discussion

For each of the patients, we performed user-centric assays in order to predict glycemia using other concurrent variables. We decided to use the data from the past 6 h as previous work indicated that this was the most relevant time slot. We decided to predict at 15 and 45 min (predictive horizon, PH). We performed sliding cross validation for model building and later predictions by shifting the time window of past data and thus the predictive horizon.

**Figure 2:**
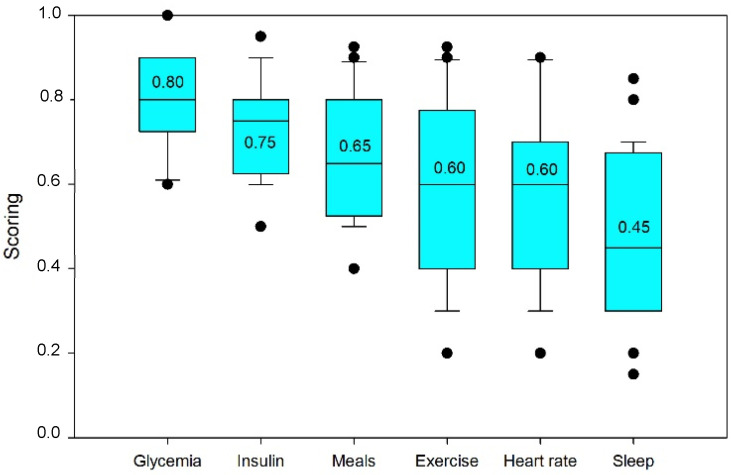
Feature selection ranking.

Before the prediction phase, variable selection was performed using LASSO in order to discuss the results of the variable selection ranking for that algorithm and historical data. Figure 2 depicts the ranking obtained.

We began by discussing the primary variable by itself, glycemia. Consequently, this will serve as the expected characteristic and need to be an inlet variable in a model for prediction (taking into account the past data).

In the ranking of importance, glycemia took the first position. Although some autoregressive models merely account for glycemia in the preceding half hour [47], these choices are not properly explained. We need to realize that this characteristic in some manner incorporates many other conditions that might effect glycemia, so we saw the last six hours as a recap of the past’s significant events.

Insulin was the second most influential feature, followed by meals. In this respect, we may comprehend that the standard deviation for insulin was less, given that its behavior was more consistent among individuals.

The major action of rapid-acting insulin (boluses) typically lasts for two and a half hours, with a maximum at 90 min [48]. Nonetheless, there are indications of a longer, lingering effect. Insulin on board has appeared often in the scientific literature; it refers to the quantity of insulin that is now present in the body and, therefore, remains acting. It includes basal insulin and residual fast insulin, which may exert a low-intensity (but detectable) effect for a number of hours. It is believed to have an extraordinary impact within 8 h [49], despite the fact that another study indicates an acting range of five to eight hours [50].

The third crucial variable was the meals. In this instance, the effect seemed to be more varied from patient to patient, which makes sense given that the nutritional makeup and metabolism of each individual affect the absorption of food differently. Thus, a meal with a higher amount of fat will alter glycemia later [51,52]; conversely, the consumption of fiber might delay the absorption of carbs [53].

Inasmuch as exercise raises glucose demand and insulin sensitivity, its effect on the evolution of glycemia has been investigated. It is highly correlated with heart rate, and because of their high correlation, exercise data could be excluded because it can be considered as included in the heart rate time series; however, this consideration must be made with caution and is left open for consideration, depending on the dataset, patients, and their habits.

This outcome was consistent with the literature on sports influence [54], in that it encompasses a broad variety of sports and intensities and exhibits a high degree of variation. Regardless, exercise continues to have an impact up to 48 h afterwards [55]. According to the data, we could differentiate between a group of individuals who engaged in high-to-moderate intensity sports and a minority who engaged in physical activities with less intensity.

Heartbeat was variable number six. If an elevated heart rate is not the consequence of physical exercise, it may be due to stress [56]. Moreover, variations in heart rate may also be symptomatic of hypoglycemia [57]. Thus, heart rate responses vary considerably among people.

Sleep was the last feature taken into account. In light of the fact that a lack of sleep is associated with a rise in hyperglycemic stress hormones, the effect of the number of hours spent sleeping was explored. Although having a low influence and being pretty variable from person to person, sleep deprivation has been pointed as a cause of insulin resistance [58], resulting in hyperglycemia in diabetic people.

We analyzed the results of the forecasting stage. These results are shown in Table 3.

**Figure 3:**
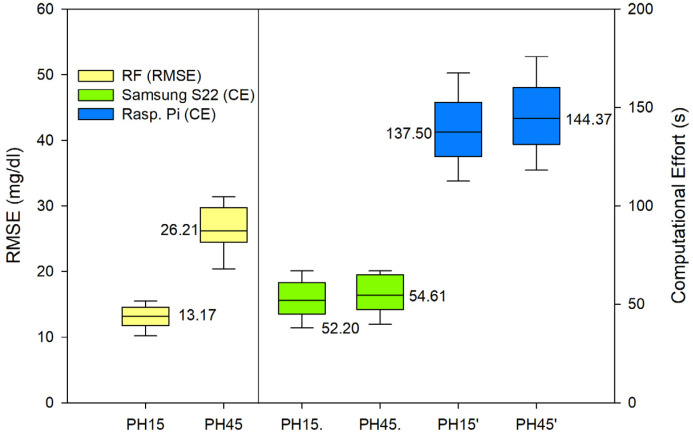
RMSE for RF predictions. PH 15 min and 45 min. CE of both PH executed in smartphone and Raspberry Pi.

**Figure 4:**
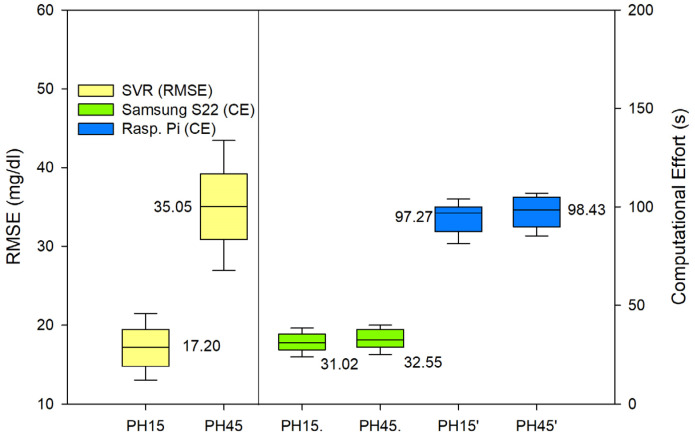
RMSE for SVR predictions. PH 15 min and 45 min. CE of both PH executed in smartphone and Raspberry Pi.

In order to evaluate the performance of the random forest (RF) and support vector regression (SVR) models for predicting glycemic levels in patients with type 1 diabetes mellitus, the root mean square error (RMSE) was calculated for both models. The RMSE values were then graphically displayed in Figure 3 for the RF model and Figure 4 for the SVR model. The RMSE values were calculated in the same manner for both models, and the results were replicated equally for both PH on the Samsung S22 and the Raspberry Pi. Additionally, to assess the computational efficiency of the models, the time required for the model to perform the prediction calculations was recorded and presented as seconds taken in the respective figures. The results of these analyses provide valuable insight into the performance of the models and their computational efficiency, which can be used to inform future research and development efforts in the field of type 1 diabetes mellitus management.

**Figure 5:**
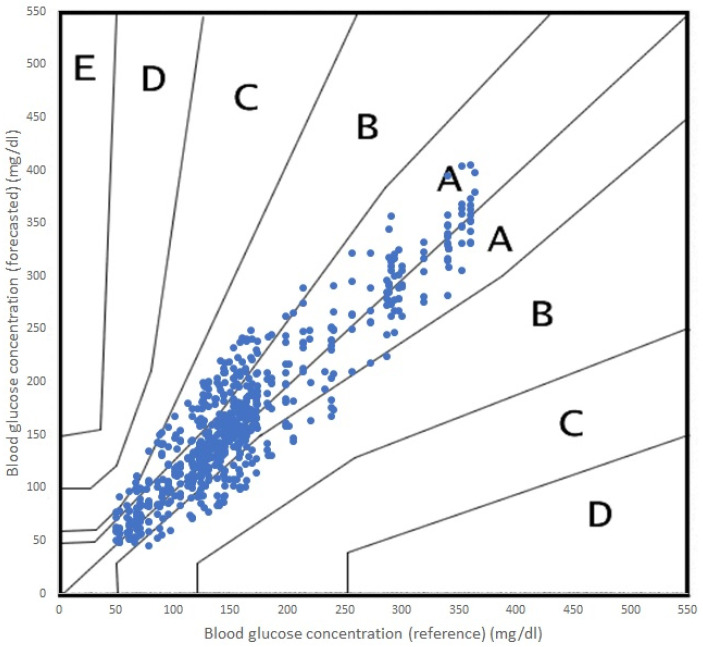
Parkes error grid for RF predictions, PH 45 min.

**Figure 6:**
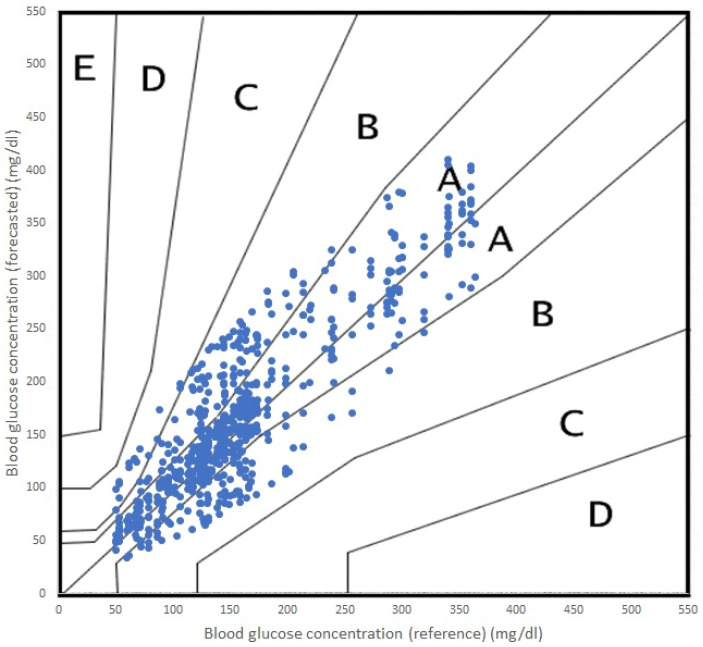
Parkes error grid for RF predictions, PH 45 min.

In terms of accuracy, both algorithms performed adequately, with acceptable errors for both RF and SVR. It is true that RF performed a more accurate prediction and SVR offered a poorer prediction for the 45 min PH. In terms of the Parkes error grid [59], the performance of both algorithms are shown in Figure 5 (RF) and Figure 6 (SVR). The grid is divided into five zones, A through E, each representing a different level of clinical risk:

- Zone A: This zone represents the most accurate predictions, where the predicted blood glucose values are very close to the reference values. Predictions in this zone are considered clinically acceptable and would lead to appropriate treatment decisions.
- Zone B: Predictions in this zone are also considered clinically acceptable, although they are less accurate than those in Zone A. The differences between predicted and reference values in Zone B may result in minor changes to treatment decisions but would not pose any significant risk to the patient.
- Zone C: In this zone, the predictions deviate further from the reference values and may lead to unnecessary treatment adjustments. While these discrepancies might not cause immediate harm to the patient, they could result in suboptimal diabetes management.
- Zone D: Predictions in Zone D are considered potentially dangerous, as they could lead to incorrect treatment decisions that may cause harm to the patient. For example, a prediction in this zone might cause a patient to administer insulin when it is not needed, resulting in hypoglycemia.
- Zone E: This zone represents the most inaccurate and dangerous predictions, where the predicted values are entirely opposite to the reference values. Predictions in this zone would lead to severe treatment errors that could have life-threatening consequences for the patient.

As we can see in Figure 6, the incursions in Zone C were more numerous.

The weight of a machine learning algorithm depends on several factors, such as the size of the training data, the complexity of the model, and the computational resources required to fit and use the model. In general, both support vector regression (SVR) and random forest can be considered “heavy” algorithms compared to some simpler algorithms, such as linear regression or k-nearest neighbors.

In terms of computational efficiency, random forest is typically considered to be a heavier algorithm compared to support vector regression [60]. This is because random forest builds multiple decision trees and combines the predictions of these trees to make the final prediction. The computational cost of building these trees can be significant, especially for large datasets. On the other hand, support vector regression is a relatively simple algorithm that requires relatively low computational resources, making it more computationally efficient compared to random forest.

In general, SVR can be considered a relatively lightweight algorithm compared to random forest, especially for small to medium-sized datasets. SVR has a simple model structure, and the optimization problem can be formulated as either a linear or non-linear optimization problem, depending on the choice of kernel.

On the other hand, random forest is a more complex algorithm that can require more computational resources, especially for large datasets. Random forest builds multiple decision trees and combines their predictions to make the final prediction. This can be computationally intensive, especially for large datasets, as each decision tree requires time and computational resources to train.

The RF computational effort on a Raspberry Pi was at the limit of what was acceptable, having average times that fell within the tolerable range considering that the sampling frequency is 5 min. However, if we want to ensure that the device will be able to make a prediction before the next data arrives, we should either run SVR or RF, but the latter should be limited to running on the Samsung S22.

## 7. Conclusions

When comparing machine learning algorithms for forecasting, it is essential to consider their computational requirements, as this can have a significant impact on their suitability for constrained devices. Although both random forest and support vector regression (SVR) can produce acceptable forecasting results, the actual computational requirements will depend on the specific characteristics of the machine learning problem being addressed.

For small datasets, random forest may not be computationally intensive, and a powerful device may be able to handle the algorithm’s requirements with ease. On the other hand, larger datasets may require more computational resources to process and SVR may become more computationally intensive, especially if a non-linear kernel is used [61]. Therefore, for machine learning problems with large datasets or complex non-linear relationships, SVR may not be the best choice for constrained devices.

Despite this, random forest can provide highly accurate predictions for problems with complex and non-linear relationships between inputs and outputs. The algorithm is well-suited for handling noisy and non-linear data and can handle high-dimensional input problems. In many cases, constrained devices can handle random forest-based forecasting tasks with some limitations.

In conclusion, when selecting a machine learning algorithm for forecasting, it is essential to consider the specific characteristics of the problem being addressed and the computational requirements of each algorithm. While both random forest and SVR can produce acceptable forecasting results, the actual computational requirements will depend on the specific problem characteristics and the suitability of each algorithm for constrained devices will vary accordingly. With careful consideration and optimization, however, limited devices can still perform machine learning tasks such as forecasting effectively, despite their computational constraints.

The present study has taken into account the measurements of 40 diabetics, which may be a limitation given the characteristics of the volunteers. Although our sample reflected a wide range of glycemic control, it is possible that patients who experience very poor disease care may suffer from poor accuracy performance.

One potential future direction for the proposed method is to explore the feasibility of incorporating real-time physiological data on the fly in the predictive model. This could lead to more accurate and personalized glucose level predictions, which could facilitate more effective diabetes management.

Another direction is to investigate the potential of integrating the proposed method with closed-loop insulin delivery systems, such as artificial pancreas devices, to create a closed-loop system that can automatically adjust insulin dosages based on predicted glucose levels. This could improve diabetes management by reducing the risk of hypo- and hyperglycemia and potentially enhance patient quality of life.

Additionally, future research could explore the potential of applying the proposed method to other types of diabetes, such as type 2 diabetes, and investigate its generalizability to other populations, including pediatric and elderly patients.

## Figures and Tables

**Table 1:** Hyper-tunning parameters.

Algorithm	Parameter	Range
Random Forest (RF)	Max depth	10 to 70
	Min samples leaf	1 to 4
	Min samples split	2 to 10
	n estimators	200 to 1200
Support Vector Regression (SVR)	C	0.1 to 1000
	gamma	1 to 0.0001
	kernel	‘rbf’

**Table 2:** Information on the DM1 patients included in the trial.

Features	Value		
Number of patients	40		
Sex	24 men–16 women		
Population Characteristics	Median	Min	Max
Age (years)	22.53	18	56
Body Mass Index (BMI, kg/m^2^)	21.30	18.25	23.71
Duration of diabetes (years)	12	4	29
HbA1C (%)	6.7	6.1	7.8

**Table 3:** RMSE and CE according to algorithm and PH.

Algorithm	PH	RMSE (mg/dL)	Device/CE (s)	
			Samsung S22	Raspberry Pi
RF	15 min	13.17	52.20	137.50
RF	45 min	26.21	54.61	144.37
SVR	15 min	17.20	31.02	97.27
SVR	45 min	35.05	32.55	98.43

## Data Availability

Data may be made available upon request to the corresponding author, subject to certain personal data protection restrictions.
